# Annotating genomes with massive-scale RNA sequencing

**DOI:** 10.1186/gb-2008-9-12-r175

**Published:** 2008-12-16

**Authors:** France Denoeud, Jean-Marc Aury, Corinne Da Silva, Benjamin Noel, Odile Rogier, Massimo Delledonne, Michele Morgante, Giorgio Valle, Patrick Wincker, Claude Scarpelli, Olivier Jaillon, François Artiguenave

**Affiliations:** 1 CEA, DSV, Institut de Génomique, Genoscope, 2 rue Gaston Crémieux, CP5706, 91057 Evry, France; 2 CNRS, UMR 8030, 2 rue Gaston Crémieux, CP5706, 91057 Evry, France; 3 Université d'Evry, 91057 Evry, France; 4 Scientific and Technology Department, strada le Grazie 15, 37134 Verona, Italy; 5 Istituto di Genomica Applicata, Parco Scientifico e Tecnologico di Udine, Via Linussio 51, 33100 Udine, Italy; 6 CRIBI, Università degli Studi di Padova, viale G. Colombo, 35121 Padova, Italy

## Abstract

Next generation technologies enable massive-scale cDNA sequencing (so-called RNA-Seq). Mainly because of the difficulty of aligning short reads on exon-exon junctions, no attempts have been made so far to use RNA-Seq for building gene models *de novo*, that is, in the absence of a set of known genes and/or splicing events. We present *G-Mo.R-Se *(Gene Modelling using RNA-Seq), an approach aimed at building gene models directly from RNA-Seq and demonstrate its utility on the grapevine genome.

## Abstract 2

A method for de novo genome annotation using high-throughput cDNA sequencing data.

## Background

Next generation sequencing technologies generate many short reads of DNA fragments in a reduced time scale and have lowered the cost per nucleotide [1,2]. Genomic short reads have been used to investigate genetic variation [3], genomic rearrangements [4], DNA methylation [5], and transcription factor binding sites (Chip-Seq) [6,7]. New algorithms had to be developed for genome resequencing, in order to map very high numbers of reads efficiently [8-11], as well as for *de novo *genome assemblies, in order to cope with the short length of reads (usually less than 35 nucleotides) [12-16]. The next-generation sequencing methods have also been applied to sequence cDNAs rather than genomic DNA, in order to catalogue microRNAs [17-19] or analyze the transcriptional landscape of a number of eukaryotic genomes: this technology is called RNA-Seq [20-26].

Before the development of the RNA-Seq technology, large-scale RNA analysis could be performed with two types of approaches. The first, tag-based approaches [27], such as serial analysis of gene expression (SAGE) [28] and massively parallel signature sequencing (MPSS) [29], were based on the sequencing of previously cloned tags located in specific transcript locations (usually 3' or 5' ends). Transcript abundance could be derived from tag counts in already known loci, but no new genes or new alternative splice forms could be discovered. The alternative approach, hybridization-based microarrays, has the potential of monitoring the expression level on the whole transcriptome (not necessarily biased towards known genes, when using whole genome tiling arrays [30-32]) at low cost, but it is biased by the background levels of hybridization and the fact that probes differ in their hybridization properties. Nevertheless, the gold standard method for transcript discovery remains expressed sequence tag (EST) sequencing (by Sanger technology) of cloned cDNAs [33-35]. Its main limitation, in addition to the relatively high cost, is that this method is sensitive to cloning biases. The RNA-Seq technology combines the advantages of the previous large-scale RNA analysis methods by enabling the monitoring of the transcriptional landscape of a whole genome at low cost, without the biases introduced by arrays, and has the additional advantage of providing information on the transcript structures (exon-exon boundaries), as EST Sanger type sequencing does on a longer range, but without cloning biases. Moreover, because a large number of reads can easily be obtained, RNA-Seq is sensitive enough to detect transcription for genes with low expression levels, which are usually missed by EST analysis [21,23,25].

In recent studies, RNA-Seq has mainly been used to quantify the expression levels of already annotated loci, identify differentially expressed genes, and measure expression outside of those loci (in intronic or intergenic regions) [21-24,26]. Additionally, structural information has been used to detect already known alternative splice forms [22,23], identify new transcriptional events in relation to known loci (alternative splicing, 5' ends) [24,26], and refine annotated gene structures or propose novel gene models [21,23]. However, no attempts have been made to take advantage of the connectivity information contained in RNA-Seq data for building gene models *de novo*, that is, in the absence of a set of known genes and/or splicing events.

Traditionally, EST, cDNA and protein sequences are the most accurate resource for identifying gene loci and annotating the exon/intron structure on genomic sequences [36]. These resources can be mapped on a genomic sequence with a global alignment strategy that allows gap insertions of genomic regions corresponding to potential introns bordered by splice sites [37-41]. The resulting positions of exon and intron boundaries can then be assembled to build complete transcript structures [42]. But the methods used to build spliced alignments of ESTs on genomes are not applicable to short reads, since they require that the sequence blocks surrounding a splice junction are long enough and highly similar to the genomic region in order to build a non-ambiguous alignment covering the exon-exon boundary. New methods are now emerging for building spliced alignments of short sequence reads [43]. However, they still require *a priori *information about the genome analyzed (splice site characteristics) in order to reduce the number of junctions to test, since testing all possible 'GT/C-AG' pairs in a genome is obviously unfeasible.

In this study, we present a method aimed at using RNA-Seq short reads to build *de novo *gene models. First, candidate exons are built directly from the positions of the reads mapped on the genome (without *ab initio *assembly of the reads), and then all possible splice junctions between those exons are tested against unmapped reads: the testing of junctions is directed by the information available in the RNA-Seq dataset rather than by *a priori *knowledge about the genome. Exons can then be chained into stranded gene models. We demonstrate the feasibility of this method, which we call *G-Mo.R-Se *(for Gene Modelling using RNA-Seq), on the grapevine genome [44] using approximately 175 million Solexa/Illumina RNA-Seq reads from four tissues. This allowed the identification of new exons (in known loci) and alternative splice forms, as well as entirely new loci. We show that this approach is an efficient alternative to standard cDNA sequencing: it detects more transcripts at lower cost. It could be particularly helpful in the case of species for which few resources are available (that is, that are very distant from the species currently present in the ESTs/protein databases). *G-Mo.R-Se *can also be combined with other data into an automatic or manual eukaryotic genome annotation. All the data described in this article are available from the *G-Mo.R-Se *website [45].

## Results and discussion

### Building gene models from RNA-Seq reads

**Figure 1:**
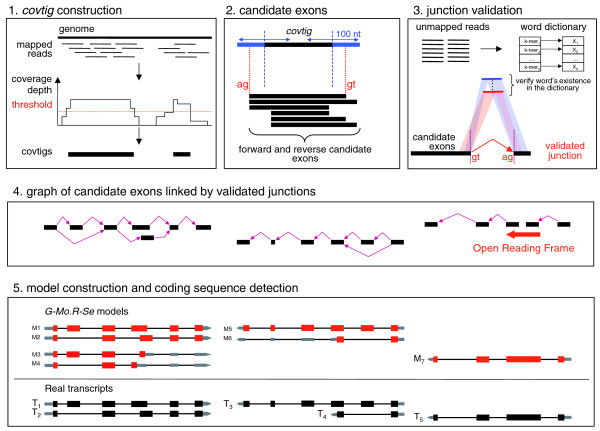
***G-Mo.R-Se *method for building gene models from short reads**. The five black boxes show the 5 steps of the approach. Step 1 (covtig construction) is the construction of covtigs (coverage contigs), which are built from positions where short reads are mapped above a given depth threshold. Step 2 (candidate exons) is the definition of a list of stranded candidate exons derived from each covtig. Splice sites are searched 100 nucleotides around each covtig boundary, which allows the orientation of the candidate exons on the forward or the reverse strand, as shown in the second box. Step 3 (junction validation) consists of the validation of junctions between candidate exons using a word dictionary built from the unmapped reads. During step 4 (graph of candidates exons linked by validated junctions), a graph is created where nodes are candidate exons (black boxes) and oriented edges (purple arrows) between two nodes represent validated junctions. The two last connected components show an example of a split gene that can be corrected using open reading frame detection between the last exon of the first model and the first exon of the second model. In the final step, step 5 (model construction and coding sequence detection) we go through the previous graph and extract all possible paths between each source and each sink. Each path will then represent a predicted transcript, and a CDS will be identified for each transcript. Models M_1_, M_2_, M_5 _and M_7 _(untranslated regions are in grey, introns in black and coding exons in red) correctly model real transcripts T_1_, T_2_, T_3 _and T_5 _(untranslated regions are in grey, and introns and exons are indicated by black lines and boxes, respectively). As all possible paths are extracted from the graph, some of them may not correspond to real transcripts (for example, models M_3_, M_4 _and M_6_).

We obtained 173 million Solexa/Illumina RNA-Seq reads from mRNAs extracted from four tissues (leaf, root, stem, callus). Of these, 138 million reads could be mapped unambiguously with SOAP (Short Oligonucleotide Analysis Package) [8] to the *Vitis vinifera *genome sequence assembly [44]. The mapped reads were contiged to build candidate exons, which we call 'covtigs' (for coverage contigs, that is, regions obtained by contiging adjacent positions with coverage depth greater than a threshold). Candidate junctions between covtigs were then tested using the unmapped reads. Finally, a graph approach was used to chain the exons through validated junctions into gene models (see Materials and methods; Figure 1). All possible chainings between exons were retained, which allowed the annotation of alternative splice forms. The covtigs that were not involved in any validated junction were discarded, implying that no mono-exonic transcripts were annotated. The procedure, which we named *G-Mo.R-Se*, produced 46,062 transcript models, clustered in 19,486 loci (an average of 2.4 transcripts per locus). A plausible coding sequence (CDS) was found for 28,399 models, clustered in 12,341 loci.

Covtig definition was essential for the subsequent testing of junctions to be efficient, especially with respect to the splits and fusions of exons (see Materials and methods). The splitting of exons into separate covtigs can occur when the read coverage depth goes down (below the depth threshold used for building covtigs), which can be due either to repeated regions (we only retained the reads that mapped at a unique position on the genome), to mismatches/gaps in the genomic sequence (we only kept the reads mapped with at most two mismatches and no indels), or to experimental biases leading to depth variations in the cDNAs sequenced and to non-normalization of the library. Indeed, some biases in the coverage uniformity of reads have been observed in previous RNA-Seq studies [23].

We aimed at correcting the splits in two ways. First, at the covtig definition step (step 1 in Figure 1), we extended the covtigs using all 16-mers found in the reads, in order to step over mismatches and short repeats. Then, at the model building step (step 4 in Figure 1), we fused together models that were linked by an open reading frame.

The artifactual fusing of exons into one single covtig can occur when the mRNA sample contains immature transcripts with retained introns, providing reads that map into the introns. Since the immature transcripts are expected to be under-represented in the set of mRNAs, the depth in the retained introns is expected to be lower than in the adjacent exons: setting an appropriate depth threshold for the building of covtigs should avoid such fusions.

The depth threshold used for covtig construction was set to balance the number of splits and the number of fusions. Indeed, low thresholds will generate few splits but numerous fusions, and conversely, high thresholds will generate few fusions but numerous splits. In order to correct more fusions, we could extend the testing of junctions inside the covtigs, instead of testing junctions only between covtigs.

We evaluated the direct mapping of reads, the initial candidate exons (covtigs), and the final models produced by *G-Mo.R-Se *at the nucleotide level in comparison to the reference *V. vinifera *annotation [44] (Table 1). The depth threshold set to build the covtigs discards most of the noise (63% of the nucleotides covered by reads are located in intergenic or intronic compartments compared to only 40% of the nucleotides covered by covtigs) while retaining the signal falling in exons (66% of the exonic nucleotides are covered by reads, and 56% are covered by covtigs). This noise is likely to correspond to transcriptional background, expression of transposable elements, or genomic contamination in the samples sequenced, rather than to SOAP mapping artifacts, since we only retained positions where reads could be mapped uniquely, with at most two mismatches. When considering final models instead of initial covtigs, the sensitivity decreases slightly (from 56% to 43% of exonic bases covered) but the specificity increases greatly (from 60% to 80% of the nucleotides - in covtigs or models - fall in the exonic compartment), suggesting that most of the covtigs that could not be linked to any other covtig resulted from transcriptional or experimental noise. The models still include about 1% of the nucleotides from the intergenic compartment, indicating that this compartment harbors new, previously unannotated, genes.

**Table 1:** Nucleotidic overlap of RNA-Seq reads, *G-Mo.R-Se *covtigs and *G-Mo.R-Se *models with different genomic compartments relative to the reference annotation

	Genomic compartment relative to the reference annotation (%)					
	
	Exonic: 41,603,635 nucleotides		Intronic: 184,047,761 nucleotides		Intergenic: 271,857,375 nucleotides	
			
	Specificity	Sensitivity	Specificity	Sensitivity	Specificity	Sensitivity
Reads: 73,580,625 nucleotides	37	66	40	16	23	6
Covtigs: 38,484,212 nucleotides	60	56	20	4	20	3
Models: 22,213,316 nucleotides	80	43	5	1	15	1

Specificity reflects the percentage of nucleotides in reads/covtigs/models falling in the compartment; sensitivity reflects the percentage of nucleotides in the genomic compartment overlapped by reads/covtigs/models.

We managed to select a satisfying depth threshold with respect to the splits/fusions (Figure S1 in Additional data file 1), as well as the signal/noise ratios. Obviously, the optimal depth threshold will be highly dependent on the characteristics of the dataset analyzed, such as the complexity of the transcriptome, the amount of alternative splicing, the amount of transcription outside of protein-coding genes, and the sequencing depth, and must be carefully selected in order for *G-Mo.R-Se *to work optimally.

### Comparing the *G-Mo.R-Se *pipeline with direct assembly of reads

**Figure 2:**
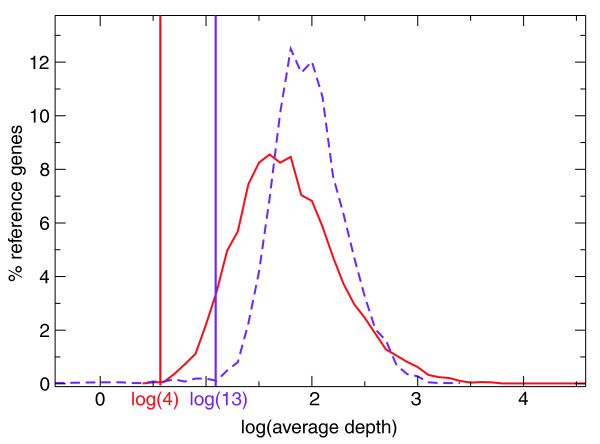
**Read coverage depth for reference genes overlapped by *G-Mo.R-Se *models and Velvet contigs**. The distribution of the average depth (log) on all exonic nucleotides of the genes is plotted for genes overlapped on ≥ 75% of their nucleotides by *G-Mo.R-Se *models (red line) and Velvet contig (dashed purple line). The y-axis corresponds to the percentage of reference genes in each bin (bin width is 0.2).

We compared the final *G-Mo.R-Se *models and the structures obtained by assembling the reads with Velvet [14] and mapping the assembled contigs to the genome with est2genome [37] (Table 2). Fewer reference genes are overlapped (on at least one nucleotide) by spliced Velvet contigs than by models (40.3% and 50.3%, respectively). The number of genes overlapped on at least 75% of their nucleotides drops even more for Velvet contigs compared to *G-Mo.R-Se *models (from 30.6% to 11.8%), indicating that most of the genes that are overlapped by Velvet contigs are not covered over their whole length. The average number of models or Velvet contigs per gene - 1.28 and 2.05, respectively - also reflects that the reference genes are more fragmented by Velvet contigs than by *G-Mo.R-Se *models. Additionally, we investigated the accuracy of the *G-Mo.R-Se *models and Velvet contigs on the structural point of view using a collection of cDNAs: 56% of the cDNA loci are predicted exactly (all exon/intron boundaries) by *G-Mo.R-Se *models, and 32% by Velvet contigs (Table S1 in Additional data file 1). We compared the average coverage depth of reference genes that are correctly annotated by *G-Mo.R-Se *models and Velvet contigs (that is, that have at least 75% of their nucleotides covered). A minimal depth of 4 is sufficient for *G-Mo.R-Se *models to annotate genes satisfactorily, whereas a minimal depth of 13 is required for Velvet contigs (Figure 2). Since *G-Mo.R-Se *relies on the genome sequence, no significant overlap between reads is necessary to put them together in a covtig: they just need to be adjacent on the genome. This explains why a much lower coverage depth is required for *G-Mo.R-Se *than for Velvet. Unlike direct assembly of reads, the *G-Mo.R-Se *pipeline is able to detect transcripts that are weakly represented in the reads set (either because they are weakly expressed or problematic to extract).

**Table 2:** Overlap of the 30,434 reference genes with Velvet spliced contigs and *G-Mo.R-Se *models

	Velvet assembly + mapping	*G-Mo.R-Se *models
Percentage of reference exonic nucleotides covered	24.8%	42.9%
Reference genes overlapped on ≥ 1 nucleotide	12,270 (40.3%)	15,323 (50.3%)
Reference genes overlapped on ≥ 75% nucleotides	3,595 (11.8%)	9,306 (30.6%)

### Comparing the *G-Mo.R-Se *approach to a classic cDNA sequencing approach

We compared the *G-Mo.R-Se *pipeline to a classic cDNA sequencing approach, using a reference set of 112,175 *V. vinifera *cDNA sequences from five tissues (including 87,199 multi-exonic cDNAs clustered in 7,895 loci) that were sequenced with the Sanger technology during the course of the *V. vinifera *genome sequencing and annotation project [44] (Table 3).

**Table 3:** Overlap of cDNA loci (all loci and loci where all 32-mers are unique) with *G-Mo.R-Se *models

	All cDNA clusters (7,895)	cDNAs clusters where all 32-mers are unique (4,822)
Percentage of cDNA (exonic) nucleotides covered by models	76.0%	87.2%
cDNA clusters overlapped on ≥ 1 nucleotide by models	6,831 (87%)	4,581 (95%)
cDNA clusters overlapped on ≥ 75% nucleotides by models	5,449 (69%)	3,997 (83%)

**Figure 3:**
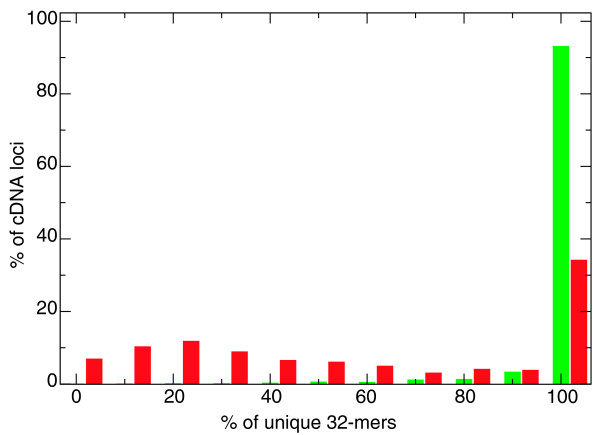
**Proportion of unique 32-mers in cDNA clusters**. The percentage of unique 32-mers is shown for cDNA clusters overlapped by models on more than 75% of their nucleotides (green) and cDNA clusters not overlapped by models (red). The y-axis corresponds to the percentage of cDNA clusters in each bin (bin width is 10% of unique 32-mers among all 32-mers in the cluster).

The 46,062 *G-Mo.R-Se *models overlap about 70% of the 7,895 cDNA loci (on more than 75% of their nucleotides). The most obvious reason why about 15% of the cDNA loci are not overlapped by any model is that they correspond to repetitive DNA. We compared the proportion of unique 32-mers (in the whole *V. vinifera *genome) for the 5,449 cDNA loci well covered by models and the 1,064 cDNA loci uncovered by models. It appears that most of the cDNA loci that were missed by models are mainly constituted of non-unique 32-mers (Figure 3). When considering only the 4,822 loci where all the 32-mers are unique, 95% of the cDNA loci are hit by a model (Table 3). Among the 5% of cDNA loci that are missed, some are too poorly covered by reads for covtigs to be built and/or junctions to be validated, and others have reads in their introns, which create fused exons, preventing the models from being detected as spliced, since one large covtig spans the whole locus.

**Figure 4:**
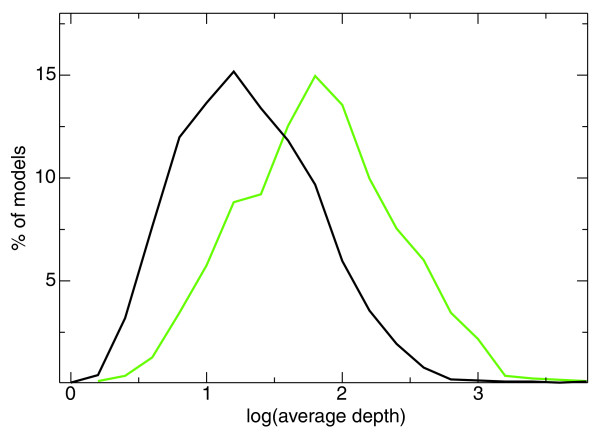
**Read coverage depth for models overlapping cDNA loci and models not overlapping cDNAs**. The distribution of the average depth (log) on all exonic nucleotides of the models is plotted for models overlapping cDNAs on ≥ 50% of their nucleotides (green) and models not overlapping cDNAs (black). The y-axis corresponds to the percentage of models in each bin (bin width is 0.2).

Interestingly, *G-Mo.R-Se *detects 2.5 times as many loci as the standard cDNA sequencing approach (19,486 loci versus 7,895). Among the 19,486 *G-Mo.R-Se *loci, only 36% overlap cDNA loci. We compared the characteristics of the 5,698 *G-Mo.R-Se *loci that overlap cDNAs on at least 50% of their nucleotides and the 12,392 loci that are outside cDNA loci (Figure 4). The *G-Mo.R-Se *loci that are new with respect to standard cDNAs tend to be expressed at lower levels than the loci that overlap cDNAs. These loci are investigated in more detail in the section 'Identifying novel genes and improving gene annotation'. The RNA-Seq technology, combined with *G-Mo.R-Se*, is able to detect gene expression that would be scored silent with a standard cDNA cloning and sequencing approach, or would necessitate an extensive Sanger sequencing effort.

On average, we annotated 2.4 models per locus. By removing the redundancy (structures fully included in other structures; see Materials and methods) from the cDNA sequences, we retained 9,827 representative sequences, with an average of 1.25 transcripts per locus. The models appear to be capturing more alternative splice forms than the cDNAs. However, as we build all possible models that correspond to the longest possible paths going from one covtig to another through validated junctions, some of the models probably do not correspond to real transcripts (for instance, if they link alternative exons that are incompatible, like models M_3 _and M_4 _in Figure 1). Since the long-range splice contiguity can not be inferred from short reads, we quantified short-range alternative splicing events in the models (all models, and only CDS portions of coding models) and in the cDNAs [46] (Table 4).

**Table 4:** Alternative splicing events detected in cDNAs, all *G-Mo.R-Se *models, and CDS portions of *G*-*Mo.R-Se *models

	cDNAs: 7,895 loci	Models (all): 19,486 loci	Models (CDS): 12,341 loci		
					
	Number (%)	Number (%)	Number (%)	Events common to cDNAs and models	% of cDNA events
Alternative acceptor/donor	690 (73.1%)	7,405 (62.5%)	2,988 (58.0%)	156	22.6
Skipped	250 (26.5%)	3,656 (30.9%)	1,677 (32.5%)	18	7.2
Mutually exclusive	4 (0.4%)	781 (6.6%)	487 (9.5%)	1	25.0
Intron retention	1,227	-	-	-	-
Total	2,171 (944 without IR)	11,842	5,152	175	18.5
Total number of loci with alternative splicing (% of all identified loci)	783 (9.9%) (598 without IR)	1,602 (8.2%)	1,029 (8.3%)	-	-

**Figure 5:**
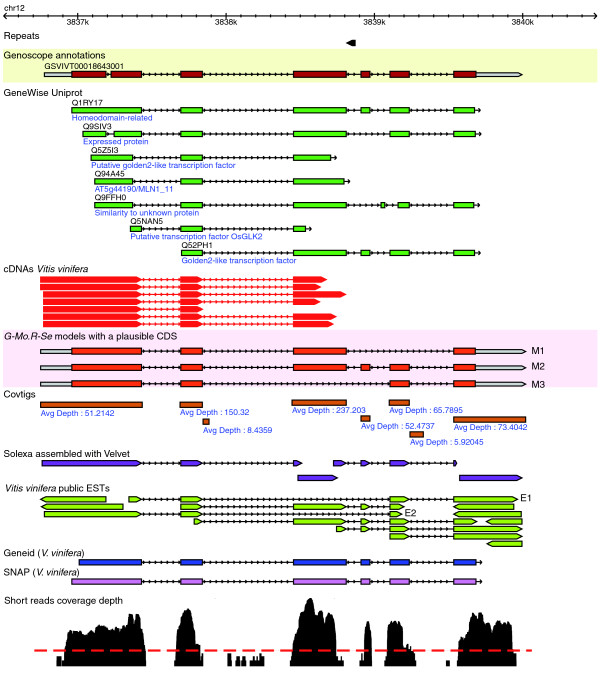
**Example of alternatively spliced models built from short reads**. The figure shows a capture of a 4 kb genomic region from *V. vinifera *chromosome 12 between 3,836,500 bp and 3,840,500 bp. The first track (Genoscope annotations) contains the automatic annotation from [44]. The green models are GeneWise alignments of Uniprot proteins. Alignment of *V. vinifera *cDNAs from [44] are in red, and public *V. vinifera *ESTs are in light green. The next track displays the models predicted by *G-Mo.R-Se *(untranslated region in grey, CDS in red). Initial covtigs are displayed as brown boxes (average depth of covtigs is written below each covtig). Alignments of velvet contigs are displayed in purple. *Ab initio *models produced by geneID [51] and SNAP [52] are displayed in blue and pink, respectively. The short reads coverage depth is plotted on the last track (black): the dashed red line shows the threshold used to build covtigs. Model M_2 _is confirmed by numerous resources, model M_3 _seems to be a minor alternative splice form (it is only supported by two public ESTs: E_1 _and E_2_), and model M_1 _is a novel alternative splice form.

The *G-Mo.R-Se *pipeline does not allow the detection of intron retentions (IRs), since we do not currently test junctions inside covtigs: if the depth in the retained intron is greater than the threshold we used to build the covtigs, we will get only one splice variant containing the retained intron. It is likely that most of the exon fusions we detected by comparison with the cDNAs (Figure S1 in Additional data file 1) correspond to cases of IRs. However, we were able to detect alternative donors or acceptors, skipped exons, and mutually exclusive exons. The relative abundance of these different classes of events is similar in the models and the cDNAs (from the most prevalent to the least prevalent: alternative acceptors/donors, skipped exons, mutually exclusive exons), but the total number of alternative splicing events in models (11,842 in all models, 5,152 in CDS portions) is much higher than in cDNAs (944 events, when removing the 1,227 IRs). The splice forms expressed at low levels, which could not be detected with cDNA cloning and Sanger sequencing, appear to harbor an unexpected number of alternative splicing events. It is likely that all these events are not compatible with the coding capacity of the transcripts. However, when restraining the analysis to the coding portions of models with plausible CDSs (that is, likely to be correctly predicted), the number of alternative splicing events remains higher than for cDNAs and the proportions of the different types of events remain unchanged. As an example, Figure 5 shows a locus where three alternative coding models were predicted: two of them (M_2 _and M_3_) are already supported by EST evidence, but the third model (M_1_) corresponds to a novel alternative splice form. Although the number of alternative splicing events is higher in the RNA-Seq dataset than in the cDNA dataset, the proportion of loci where alternative splicing occurs is similar for cDNA clusters and *G-Mo.R-Se *models (10% and 8%, respectively). These results are in agreement with previous studies that showed that the fraction of alternatively spliced genes is lower in plants than in animals [47]. Notably, of the 944 non-IR events detected in cDNAs, the models detect only 175 (18.5%): though some of these events might result from incorrect mapping of the cDNAs, most of them are likely to be real, and to have been missed by *G-Mo.R-Se *(Table 5). The pipeline detected only 7.2% of the skipped exons and 25% of the mutually exclusive exons, which is likely due to the limited number of neighboring covtigs (20) we tested to validate the junctions. Only 22.6% of the alternative donors/acceptors were detected because we searched for junctions only 100 nucleotides around the covtig boundaries, which limited the window where alternative splice sites could be discovered (see Materials and methods). Obviously, the model construction was not designed to capture the whole alternative splicing landscape of a genome. But still, the non-exhaustive view that we obtain is much richer than what could have been suspected from classic EST sequencing. In order to study alternative splicing exhaustively, which is out of the scope of this study, specific tools will need to be developed.

**Table 5:** Characteristics of known and novel *G-Mo.R-Se *models (all, and with a plausible CDS)

	Known model loci		Novel model loci	
		
	All models	Models with a plausible CDS (65%)	All models	Models with a plausible CDS (17%)
Number of loci	18,811	12,236	675	105
Number of models	45,290	28,283	772	116
Average number of models per locus	2.4	2.3	1.1	1.1
Average number of exons per model	8.2	8.9	2.3	2.9
Number of models with more than two exons	37,644 (83%)	25,428 (90%)	128 (17%)	56 (53%)

Models were clustered in loci as described in Materials and methods.

### Identifying novel genes and improving gene annotation

Expectedly, since *V. vinifera *belongs to a phylogenetic branch where a profusion of resources are available, most of the models (95%) that fall outside of cDNAs overlap the reference annotation [44], or other resources such as GeneWise hits with Uniprot proteins [39,49], and ESTs from other species (Table S2 in Additional data file 1). However, 675 models are completely novel, or 116 when considering only models with a plausible CDS. We compared the characteristics of the models that are novel and the models that are supported by evidence, which we now call 'known' models (Table 5).

The proportion of models with a plausible CDS drops when considering the novel models compared to the known models (from 65% to 17%), as well as the average number of exons per model (from 8.2 to 2.3 for all models). It is likely that some of the novel models correspond to false predictions: if one junction is validated erroneously, it will create a false two-exon model. Nevertheless, the proportion of models with more than two exons is higher in the subset of novel models having plausible CDSs (53%) compared to all novel models (17%), which suggests that at least some of them are genuine novel coding loci. In addition, the novel loci that are non-coding could either correspond to coding transcripts that were mis-annotated by the pipeline (wrong splice site generating a frameshift, models associating incompatible exons), to coding transcripts where no CDS could be detected because of frameshifts in the genomic sequence, to genuine non-coding transcripts, or to transcriptional/experimental noise (Figure 1). The structure of one of the novel models, spanning eight exons, is shown in Figure S2 in Additional data file 1. A Blast [48] search against Uniprot [49] revealed an homology to a transcription regulator from *Arabidopsis thaliana*. The homology was below the sensitivity threshold required to map proteins to the genome during the annotation process. In addition to the discovery of novel splice forms and novel loci, *G-Mo.R-Se *models enrich the reference annotation by extending (in 5' or 3') about 40% of the reference genes they hit. *G-Mo.R-Se *models thus constitute a valuable resource for improving *V. vinifera *gene annotation.

## Conclusion

In this study, we demonstrate the feasibility of building gene models *de novo*, using only RNA-Seq reads and the corresponding genomic sequence, with a relatively straightforward annotation pipeline that we call *G-Mo.R-Se*. Using a dataset of approximately 175 million Solexa reads, it could detect more loci than could be identified by cloning and sequencing approximately 120,000 cDNAs, at a cost about 20 times lower (55% of the multi-exonic genes from the annotation are overlapped by models versus only 35% by *V. vinifera *cDNAs). Especially, *G-Mo.R-Se *allowed the annotation of loci expressed at very low levels. We show that this approach efficiently deciphers real transcripts from transcriptional/experimental noise since the junction validation step removes false positive covtigs. Additionally, although it was not designed to be exhaustive in the detection of alternative splicing events, *G-Mo.R-Se *detected more alternative splice forms than the cDNA resource, with no need for *a priori *knowledge of the exon-exon junctions to test. Finally, we could also identify putative novel genes (that had been missed by the automatic annotation procedure) in a genome that is already very well annotated owing to the plethora of resources available in this phylum. We tested the *G-Mo.R-Se *pipeline with Solexa/Illumina RNA-Seq reads but it can readily accept any other type of short reads, or combine reads from different technologies.

For future genome projects, it is conceivable to think of performing the annotation using RNA-Seq runs treated with *G-Mo.R-Se *as the unique resource, provided that the tissues or cell types sampled are representative enough to drive a comprehensive annotation. This approach will be particularly valuable in phyla where few resources are available (that is, that are very distant from the species currently present in the EST/protein databases), where the expensive and time-consuming step of constructing cDNA libraries could be avoided. When other resources are available, the gene models can also be combined with other data into automatic or manual eukaryotic genome annotation pipelines.

Although the *G-Mo.R-Se *pipeline works satisfactorily on the *V. vinifera *dataset, it is still fairly simple and we can think of several refinements. First, at the moment, no mono-exonic models are produced (such models represent only 8% of annotated grape genes), but we could easily bring back the covtigs that were not linked to any other covtig by a validated junction, if they contain a CDS that exceeds a certain length. Next, at the covtig building step, instead of using a fixed depth threshold, we could adapt it to the environment: the covtigs would be built to coincide with sharp increases/decreases in depth. Such a strategy should enable the annotation of separate exons in case of IR. In order to correct even more fusions, it would also be straightforward to test candidate junctions inside the covtigs in addition to the junctions tested between covtigs. Since the scope of this study was to annotate as many genes as possible, we chose to pool together the reads from all four tissues before building the covtigs. But we could also consider building covtigs and gene models separately in different samples, in order to investigate differential expression, although to the detriment of sensitivity. A last, more elaborate refinement would be to use the depth information in order to link together only covtigs that are likely to be part of the same transcript, instead of building all models that correspond to the longest possible paths in the graph of covtigs linked by validated junctions. Such an approach would allow speculation on longer range splice contiguity, and to study more exhaustively the alternative splicing landscape.

## Materials and methods

### RNA-Seq experiments

RNA-Seq reads were obtained (as described in Del Fabbro *et al*., unpublished data) by sequencing cDNA obtained from four tissue samples with the Solexa/Illumina technology: leaf (11 lanes), root (9 lanes), callus (9 lanes), and stem (9 lanes). The mRNA molecules were purified from total RNA extractions and fragmented before cDNA synthesis (with random hexamer primers). The protocol was not strand-specific. The single-end reads obtained were 32 nucleotides long, except for 5 lanes in the callus sample, where the reads were 35 nucleotides long. The resulting 172,545,778 usable reads (5.4 Gbases) were mapped to the *V. vinifera *genome [44] using SOAP [8] with a seed length of 12 and default parameters: 138,326,238 reads (4.6 Gbases) were mapped at one unique position with at most two mismatches and no indels. As a consequence, reads that align to exon-exon junctions could not be mapped to the genomic sequence.

### Building gene models from short reads

The *G-Mo.R-Se *method for building gene models from short reads is summarized in Figure 1. The first step is the definition of covtigs (coverage contigs). They are built by contiging the positions where short reads are aligned above a certain coverage depth threshold. This threshold is a parameter that needs to be adjusted in order to balance sensitivity and specificity as well as splits and fusions. In the absence of a training set to quantify the splits and fusions, this parameter can also be optimized by maximizing the number of junctions validated in the next step. Before the subsequent testing of junctions, the covtigs were extended using all 16-mers found in short reads, in order to step over mismatches and short repeats. It is important to note that the read length limits the detection of very short exons (< 35 nucleotides).

In the next step, we searched for donor (GT or GC on the forward strand, and AG or AC on the reverse strand) and acceptor (AG on forward strand and CT on reverse strand) splice sites 100 nucleotides inside and outside each covtig boundary. This enabled us to create a list of oriented candidate exons (with putative alternative donor and/or acceptor splice sites) for each covtig.

The third step was the validation of junctions between candidate exons using unmapped reads, since reads that align to exon-exon junctions were not mapped to the genomic sequence. We tested all candidate exons derived from a given covtig with the candidate exons derived from the 20 next covtigs. All the putative junctions were tested using a word dictionary approach. The dictionary (with a word size of 25) was built using the unmapped reads. Ten words (8 nucleotides on the first exon and 17 nucleotides on the second exon, 9/16, 10/15, 11/14, 12/13, 13/12, 14/11, 15/10, 16/9, 17/8) were derived from each putative junction, and their presence in the dictionary was tested. In order to validate a junction, at least five different words need to be found in the dictionary, and the total number of occurrences of all words derived from each junction needs to be of the same order of magnitude as the average depth of the adjacent covtigs (greater than 1/10 of their average depth).

The efficiency of the junction validation procedure relies on the covtig definition step for the following reasons: only the junctions between each covtig and the 20 next covtigs are tested, meaning that if more than 20 'false' covtigs are defined between 2 'real' covtigs, the junction between the two real covtigs will not be tested; only 100 nucleotides around the covtig boundary are scanned for putative splice sites, meaning that if the covtigs are too short or too long, the correct junction will not be tested; only junctions between covtigs are tested, meaning that if a covtig corresponds to a fusion between two exons, the correct junction will not be tested, and the final model will include a retained intron. On the other hand, if an exon is split between two covtigs, no junction will be valid between those covtigs, leading to the splitting of a gene into separate models. As a consequence, in the absence of a training set (annotated genes, ESTs, and so on) to calibrate the depth threshold used for building covtigs, it is possible to optimize the threshold by maximizing the number of validated junctions. *G-Mo.R-Se *can thus be used for *de novo *annotation.

For the last step, the model construction relies on the graph of candidate exons linked by validated junctions on the same strand. The models correspond to all the longest paths linking candidate exons through validated junctions. Candidate exons that are not involved in any validated junction are discarded, implying that no mono-exonic models are produced. In order to correct potential gene splits, we fuse together adjacent models (on the same strand) that are linked by an open reading frame.

Additionally, all models produced by *G-Mo.R-Se *are searched for CDSs. When the longest CDS (if greater than 50 amino acids) spans at least two-thirds of the nucleotides of a model or the number of non-coding exons is lower than the number of coding exons, the CDS is qualified as plausible. Models with plausible CDSs are likely to correspond to protein coding genes. Plausible CDSs could be detected for about two-thirds of the models. The *G-Mo.R-Se *models can be downloaded from the *G-Mo.R-Se *website [45] and visualized on the *V. vinifera *genome browser [50].

### *G-Mo.R-Se *models and cDNA analysis (clustering, alternative splicing detection)

The same clustering procedure was applied to models and cDNA sequences aligned on the genome. We used a single linkage clustering approach, where a link between two models was created if they had a cumulated exonic overlap (on the same strand) of at least 100 nucleotides (only overlaps of at least 10 nucleotides were considered). A graph-based approach was used to resolve the single linkage clustering. Additionally, the redundancy was removed from the cDNAs by discarding all transcript structures that were fully included in longer structures. We detected all pairwise alternative splicing events between intron pairs, with the same method as described in [46]. All tandemly duplicated genes were discarded from the alternative splicing events detected, since such genes may be artificially linked by cDNA mapping as well as model construction, and would generate false alternative splice forms spanning several loci instead of one. However, it is notable that, since the pipeline builds all possible models, it will always predict the two separate correct models in addition to the incorrect joined model(s).

## Abbreviations

CDS: coding sequence; EST: expressed sequence tag; *G-Mo.R-Se*: Gene Modelling using RNA-Seq; IR: intron retention; SOAP: Short Oligonucleotide Analysis Package.

## Authors' contributions

FD performed preliminary tests, ran the pipeline and analyzed the results. JMA had the original idea of the algorithm. CDS performed the mapping of the cDNAs and analyzed alternative splicing events. BN produced the annotation of the grapevine genome. OR developed components of the cDNA mapping pipeline. RNA-Seq data were generated and provided thanks to MD, MM and GV. PW and CS produced genomics and cDNA data and assisted in data management. OJ took care of the coordination with the *V. vinifera *consortium, and contributed to the writing of the paper. FA assisted in the design of the pipeline and manuscript preparation. FD and JMA developed the current version of the software and wrote the paper. All authors read and approved the final manuscript.

## Additional data files

The following additional data are available with the online version of the paper. Additional data file 1 is a Word file containing Tables S1 and S2 and Figures S1 and S2. Table S1: cDNA transcript structures correctly predicted by *G-Mo.R-Se *and Velvet. Table S2: support (in public resources) of *G-Mo.R-Se *models that do not overlap cDNAs. Figure S1: proportions of exon fusions and exon splits obtained with different depth thresholds for the covtig construction step. Figure S2: example of a novel model.

## Supplementary Material

Additional data file 1 Table S1: cDNA transcript structures correctly predicted by *G-Mo.R-Se *and Velvet. Table S2: support (in public resources) of *G-Mo.R-Se *models that do not overlap cDNAs. Figure S1: proportions of exon fusions and exon splits obtained with different depth thresholds for the covtig construction step. Figure S2: example of a novel model. Click here for file
