# Supplementary material for: Annotating genomes with massive-scale RNA sequencing
Source: Genome Biol. 2008 Dec 16;9(12):R175. doi: 10.1186/gb-2008-9-12-r175 (PMC2646279; doi:10.1186/gb-2008-9-12-r175)
Supplement: Additional data file 1 — Table S1: cDNA transcript structures correctly predicted by G-Mo.R-Se and Velvet. Table S2: support (in public resources) of G-Mo.R-Se models that do not overlap cDNAs. Figure S1: proportions of exon fusions and exon splits obtained with different depth thresholds for the covtig construction step. Figure S2: example of a novel model. [file gb-2008-9-12-r175-S1.doc]

**Supplementary Tables**

**Table S1** : Number of transcripts (obtained by reconciliating redundant cDNAs into transcript structures) that were predicted exactly (exon/intron structure entirely included in a model) by *G-Mo.R-Se* models and Velvet assembled contigs (first line), and number of cDNA loci containing at least one correctly predicted transcript (second line).

| Transcripts and loci derived from cDNAs | *G-Mo.R-Se* | Velvet |
| --- | --- | --- |
| Transcripts with exact structure predicted (total : 9829) | 4600 (47%) | 2529 (26%) |
| Genes having at least one transcript with exact structure predicted (total: 7895) | 4407 (56%) | 2509 (32%) |

**Table S2**: Support (in public resources) of *G-Mo.R-Se* models that do not overlap cDNAs.

|  | All *G-Mo.R-Se* models | *G-Mo.R-Se* models with a plausible CDS |
| --- | --- | --- |
| Total number of loci not overlapping cDNAs | 12,392 | 7143 |
| Number of loci not overlapping cDNAs and overlapping reference annotation | 10,751 | 6767 |
| Number of loci not overlapping cDNAs and overlapping reference annotation or Uniprot proteins mapped with GeneWise | 11,170 | 6916 |
| Number of loci not overlapping cDNAs and overlapping reference annotation or Uniprot proteins mapped with GeneWise or other public ESTS from dicotyledons and vitis | 11,717 | 7038 |
| Number of loci not overlapping any evidence | 675 | 105 |

**Supplementary Figures**


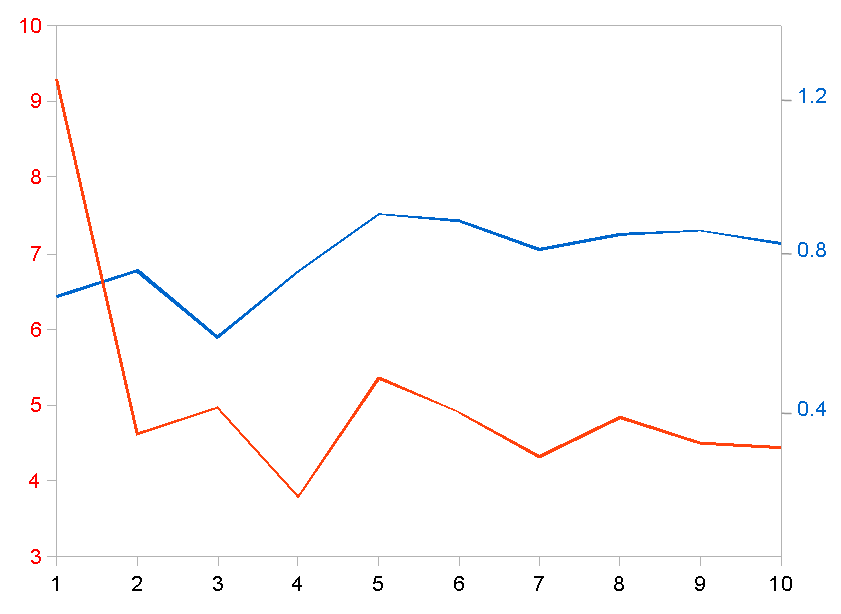


**Figure S1**: Proportions of exon fusions (red curve : % of exons from predicted models that fuse at least two reference exons, scale on the left side) and exon splits (blue curve : % of reference exons that are split by model exons, scale on the right side) obtained with different values (from 1 to 10) of depth threshold (for the *covtig* construction step). A depth threshold of 4 was retained.


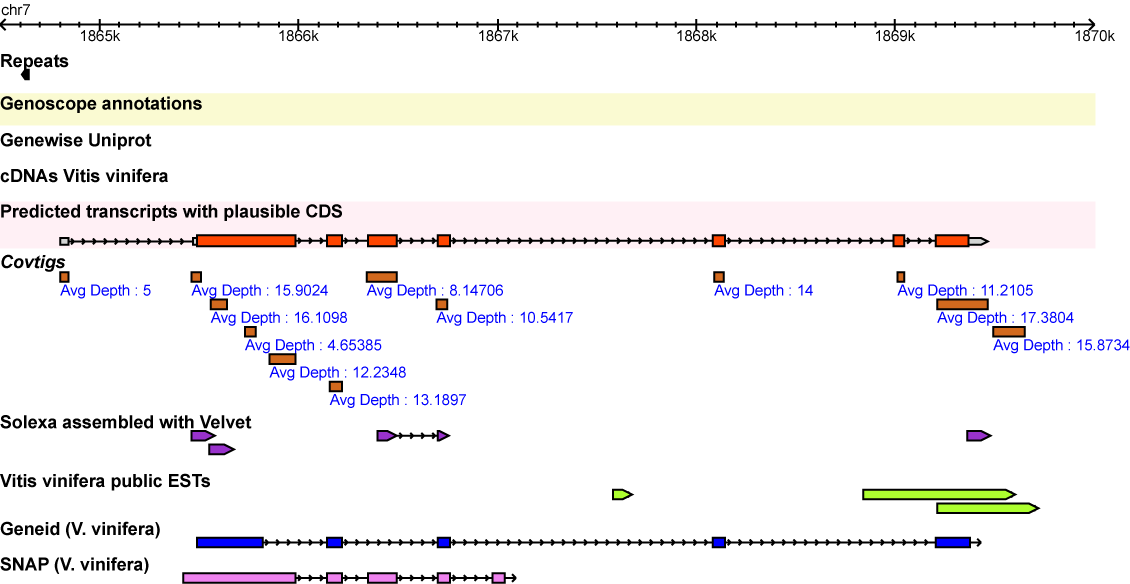


**Figure S2**: Example of a novel model spanning 8 exons, on chromosome 7, positions 1,862,136 to 1,872,135.
